# Reflections From the Pandemic: Is Connectivism the Panacea for Clinicians?

**DOI:** 10.2196/53344

**Published:** 2024-12-03

**Authors:** Jennifer Benjamin, Tyson Pillow, Heather MacNeill, Ken Masters, Anoop Agrawal, Neil Mehta

**Affiliations:** 1 Texas Children's Hospital Baylor College of Medicine Houston, TX United States; 2 Huffington Department of Education Innovation and Technology Baylor College of Medicine Houston, TX United States; 3 Department of Emergency Medicine Baylor College of Medicine Houston, TX United States; 4 Department of Medicine, Continuing Professional Development, Faculty of Medicine University of Toronto Toronto, ON Canada; 5 Medical Informatics Sultan Qaboos University Alkhod Oman; 6 Department of Internal Medicine Pediatrics Baylor College of Medicine Houston, TX United States; 7 Department of Internal Medicine Lerner College of Medicine, Cleveland Clinic Cleveland, OH United States

**Keywords:** learning theory, learning framework, connectivism, panacea, COVID-19, generative artificial intelligence, GAI, health care community, clinician, health care, airborne disease, learning, information, misinformation, autonomy, diversity

## Abstract

The COVID-19 pandemic and the recent increased interest in generative artificial intelligence (GenAI) highlight the need for interprofessional communities’ collaboration to find solutions to complex problems. A personal narrative experience of one of the authors compels us to reflect on current approaches to learning and knowledge acquisition and use solutions to the challenges posed by GenAI through social learning contexts using connectivism. We recognize the need for constructivism and experiential learning for knowledge acquisition to establish foundational understanding. We explore how connectivist approaches can enhance traditional constructivist paradigms amid rapidly changing learning environments and online communities. Learning in connectivism includes interacting with experts from other disciplines and creating nodes of accurate and accessible information while distinguishing between misinformation and accurate facts. Autonomy, connectedness, diversity, and openness are foundational for learners to thrive in this learning environment. Learning in this environment is not just acquiring new knowledge as individuals but being connected to networks of knowledge, enabling health professionals to stay current and up-to-date. Existing online communities with accessible GenAI solutions allow for the application of connectivist principles for learning and knowledge acquisition.

## Introduction

The COVID-19 pandemic was unprecedented in exposing the importance of staying current as health professional educators, the dangers of misinformation, and the need for a new approach to learning. In our commentary, we aim to highlight these 3 different aspects.

The first case of COVID-19 in the United States was confirmed in January 2020, and stay-at-home orders were not issued till March 2020 [1]. We narrate the personal experience of one of the authors during this time. The viruses’ mode of transmission and strategies to reduce spread were every health professional’s priority, and this author, a physician at a large health care system, observed that several preprint articles highlighted that transmission of infection could be potentially from airborne infection [1-5].

In March 2020, just before the COVID-19 pandemic became a reality in the United States, this author was precepting a student in an ambulatory clinic. After some discussion on the topic, the physician and the student both wore masks at the clinic. Coincidentally, another member of the team who was a member of the hospital’s COVID-19 task force walked by, commenting, “You know masks don’t work for COVID-19, right? You will more likely get infected by transferring droplets to your face as you repeatedly adjust the mask.” Due to the information in the preprint articles, the physician and student continued masking, and the physician shared the reasoning behind masking and why several respected health care organizations and experts were advocating against it.

At this hospital, the emphasis was still on droplet precaution with hand washing, thereby aligning with health organizations’ statements prioritizing the need for handwashing for droplet precautions [6,7] based on evidence-based recommendations. However, the pandemic exposed years of misunderstanding of the nature of airborne infection by the scientific community [2,8-10]. The transmission was primarily believed to be droplet and fomite-related, with airborne transmission falsely thought to occur only during aerosol-generating procedures, such as nebulizer treatments and intubations. The conventional wisdom was that particles larger than 5 microns would be transmitted as droplets rather than aerosols. This led to the assumption that most respiratory infections spread as large droplets and could be mitigated through droplet precautions.

The confusion of airborne versus droplet transmission of infection existed several decades prior with other infections, such as tuberculosis, when it was mistakenly believed that infections spread primarily as droplets, with reluctance to accept the airborne route as the cause of transmission [5]. Physicists and aerosol scientists, on the other hand, were aware of aerosol particles as large as 100 microns [11], which contradicted the 5-micron particle size distinction between aerosol and droplets [4,5,12,13]. However, the medical community was either unaware or slow to learn these facts due to the predominantly siloed approach of relying solely on previously established studies and beliefs in medical academia [3-6,12,14-17]. Health care experts in environmental sciences voiced concern about how the pandemic was being handled [3]. This physician was intrigued and, rather than dismiss the new differing opinion from environmental scientists, decided to investigate it further. As we know now, although the virus responsible for the pandemic is spread by airborne transmission, the recommendations for masking and the warning that COVID-19 was being transmitted as an aerosol were not communicated until almost 2 years later by institutions [18].

Questions to be considered at this stage are: What convinced this physician about the mode of transmission and to practice masking contrary to recommendations by their hospital or public health organizations during the initial days of the pandemic? How can a health care provider stay informed when traditional peer-reviewed literature is not yet available and recommendations from health care organizations differ from expert opinions?

As physicians, we are expected to be fully informed of the latest advances in medicine, stay connected with accurate sources of information, and, thus, make the best clinical care decisions. While the challenges posed by the pandemic may be behind us, our aim is to reflect and learn from this experience and derive solutions when there are differing opinions. Overall, the question to address is: How can one use critical appraisal to decipher signals from noise when bombarded with information?

While peer-reviewed evidence is still being generated, people often fall back on institutional guidance and policies, and one needs to recognize the need to explore differing opinions with a critical approach. With this personal experience as our background, we pivot our attention to addressing the realistic challenge of today: the role of GenAI in education. We propose the need for a different approach to learning and knowledge building to solve challenges facing health professionals with GenAI, using connectivism as a complementary approach to the tried and tested methods of constructivism. In doing this, we apply the foundational principles of learning from this personal experience to derive solutions to challenges posed by GenAI and provide concrete examples and suggestions to implement this new framework of learning.

Let us consider this physician’s approach to knowledge generation.

## Building a Knowledge Network

As the news broke of a serious respiratory illness in China, the physician created their personalized stream of information from firsthand observers, including journalists and reporters from reputable newspapers and websites, opinions of a diverse group of experts in infectious disease, virology, immunology, epidemiology, and environmental engineering by creating a list on Twitter (subsequently rebranded X). They checked the profile of experts in each field by their institutional affiliations and qualifications, ignoring follower counts and, instead, focusing on previous work using PubMed, Google Scholar, and organic web searches. Intentionally seeking diverse viewpoints, they expanded their network by adding experts in their field of interest or followed by their existing contacts. They also created an RSS feed of newly published preprint articles on the topic. By regularly perusing the knowledge network and the latest studies on the feed, they stayed current on the latest evidence-based knowledge and multidisciplinary expert opinions by being connected to new knowledge and information in real-time, thus overcoming traditional health care siloes. Their knowledge network expanded by adding authors of key studies from the RSS feed to the Twitter list. Their expanding knowledge allowed them to openly discuss evidence-based facts and highlight scientific knowledge about transmission by airborne spread in earlier studies [16]. In these discussion forums, they learned of additional articles that treated the first few US patients, suggesting multiple modalities of spread, droplet, and airborne [2]. On further inquiry, they recognized a group of environmental engineers who were elaborating on the aerosolization of large particles, traveling distances larger than the recommended 6 feet for social distancing while staying suspended in the air for long periods of time [3,4,11-13].

### Constructivism: Tried and Tested Approach to Learning

Traditionally, the dissemination of evidence-based knowledge is through information taught at medical schools, academic conferences, and peer-reviewed literature, including systematic reviews. Experts from other fields rarely teach in medical schools, and we seldom venture beyond health care literature for evidence-based information. There are few opportunities for the exchange of ideas between basic scientists and clinical researchers, which further delays the application of translational research to clinical practice. Medical training relies on years of education and learning from clinical experience. This process of building upon previous knowledge while constructing new knowledge is known as “constructivism.” Constructivism works well for established paradigms of knowledge generation at a controlled pace. However, in settings such as the pandemic and currently with GenAI, evidence is generated rapidly and often sourced from disciplines outside health care. In the example above, it was the interdisciplinary work of aerosol scientists that helped us acknowledge airborne transmission [3,12].

Constructivism takes time and is a gradual process. As individuals, we are unable to process numerous sources of information simultaneously, and technology can help address this challenge by personalizing the information we receive. When faced with new ideas that do not align with previous knowledge, we either ignore new information or work harder to reframe our understanding. Our psychological drive for consensus suppresses disagreement, sometimes even in the face of evidence-based facts [8,19]. Thus, when exposed to information contrary to previous knowledge, we fail to recognize our limitations and misunderstandings. When inundated with a flood of new research from untraditional sources, the challenge is even greater. With the acceleration of knowledge generation, it is impossible to stay informed with advances in literature. Hence, there is a need to belong to communities that amplify accurate information. Learning to distinguish misinformation from tried and tested evidence-based facts becomes an additional role for physicians. As a scientific community, we are at risk of overlooking errors and missing crucial discoveries, especially if we fail to listen to experts from other fields. We further distinguish between constructivism and connectivism in Table 1.

**Table 1:** Differentiation between constructivism and connectivism.

	Constructivism	Connectivism
Learning focus	Focuses on an individual building internal understanding	Distributed within a network through connections between information sources
Factors	Influenced by engagement, participation, and social and cultural factors	Influenced by the diversity of the network, strength of ties, reliability, and accuracy in becoming “nodes”
Process of learning	Learner builds schema based on previous understanding	Learner builds knowledge by connecting to nodes and growing network
Knowledge	Exists within an individual Connecting new information to existing knowledge	Exists outside the individual Network’s collective intelligence
Information flow	Sequential builds from a foundational understanding of simple to complex concepts	Nonlinear, influenced by strength and diversity of connections
Knowledge dissemination	Source of new information: systematic reviews and conferences, slower pace of new information	Source of new information: preprints; rapidly changing, diverse pool of resources
Technology	Supportive, enhances learning experience	Integral to learning for connections and to access information
Educator’s role	Experiential learning, helps learners construct knowledge	Learner-driven, facilitates connections, guides learners to reliable sources

### Why Consider a New Framework?

Public health institutions’ announcements from the World Health Organization and the Centers for Disease Control and Prevention reach billions of people who trust and follow their recommendations as they are based on well-founded evidence. Until fully verified, informing the public of aerosolized spread could cause panic and chaos, leading to a shortage of personal protective equipment with a huge socioeconomic impact [10]. They rely on data from multiple well-designed studies before conclusively deciding on airborne spread. Although the gold standard, this method is not completely foolproof and has its drawbacks. Many randomized controlled trials are refuted within a few years of publication, with a considerable number not published [20,21]. There are biases leading to higher publication rates of studies with positive results. Biomedical science has been in a silo, failing to learn from other disciplines and often discarding information that is obvious to other disciplines [13]. In fact, if the need for unanimity overrides motivation and authority, it can be detrimental, with negative consequences for true learning [22], and one way to combat this as a scientific community is to test and retest a hypothesis to maintain scientific rigor [23]. Overworked clinicians, stretched for time, often rely on online resources and opinions of local experts, practicing eminence-based medicine if there is insufficient evidence-based medicine. Over time, as more evidence emerges from multiple sources, the science becomes clearer, the truth emerges, and the findings are more universally adopted by physicians in practice. This process can take several years to get from the bench to the bedside, sometimes up to 5 years in surgical specialties [20].

### A New Approach to Learning: Connectivism

Connectivism was first proposed by Siemens [24] as a learning theory for a digitally connected world. In connectivism, new knowledge resides in the diversity of opinion and is distributed across networks, and learning occurs when individuals form connections across these networks [24,25]. Knowledge in a digital world where information is changing rapidly resides in several sources, experts, and networks, serving as “nodes” of information [26]. New knowledge resides outside of individuals, in digital networks and online communities [25,27]. Learning is not about acquiring new information but connecting to networks of knowledge [26]. Thus, knowing “where” and “who” instead of “what” and “how” becomes the new paradigm for information acquisition and learning. Health care experts need to be active participants in this dynamic network, serving as “nodes” that can recognize accurate information while discarding misinformation. The internet has allowed for the creation of communities and knowledge networks; learning how to navigate the diversity of opinions, differentiate reliable sources of information, and distinguish accurate facts from misinformation is, therefore, a valuable skill for future physicians. The theoretical approach to connectivism relies on the diversity of opinions, the strength of connections, the ability to recognize patterns, and the notion that the capacity to know is more important than what is already known. [25,26,28]. Coherence provides synchronization of different elements in this virtual community and provides organization and structure in the diverse networks of knowledge [29].

We consider the following implications for using connectivism as clinicians: First, the starting point of connectivism are “nodes” of accurate information; these can be individual experts, devices, or communities. Knowledge is created through interaction between the nodes and due to the diversity of opinions [26,28,30,31] by maintaining and nurturing connections and forming alliances with experts across disciplines. In these knowledge networks, Connectivism supports lifelong learning, collaboration, and critical thinking and fosters the ability to synthesize information from multiple sources [28,32]. The diversity of nodes makes it possible for networks to adopt and evolve over time [26]. The 4 foundational aspects of successful networks in connectivism are autonomy, connectedness, diversity, and openness [33].

Second, autonomy allows learners to choose their learning communities through self-directed learning in massive online open courses. Individuals have the freedom to express their ideas and explore the knowledge network to identify experts and acquire knowledge based on their level of motivation and not be restricted by a hierarchical approach to learning. Exploring the diversity of opinions allows the learner to experience the breadth of opinions. A physician with the skills to identify reliable and accurate sources of information with a diversity of opinions has a better understanding of the challenges faced by patients and can provide practical advice by directly addressing misinformation. These dialogues allow for interactivity and problem-solving [29].

Connectedness enables collaboration, and learning occurs through new connections between nodes and being connected to the right sources allows knowledge propagation in networks [29]. Maintaining, nurturing, and contributing to this network with coherence allows for continual learning, with new knowledge residing in the community rather than the individual. Connection with the right people in the right context contributes to learning. The ability to find and analyze new information quickly is crucial. With rapid changes in scientific understanding, staying current by collaborating with experts outside one’s field is essential. Currency and staying current with accurate information is the intent of connectivist learning [34].

### Connectivism and the Future Implications

Learning in connectivism is based on the production of knowledge rather than the consumption of knowledge by prioritizing relevant data while filtering out irrelevant information. In the digital environment, learning is enhanced by engagement with virtual communities of practice through blogs, posts on X, podcasts, and meaningful dialogue [35]. Interactions are central in the online educational experience, and for deep learning to occur, these interactions need to be purposeful with systematic discourse [36]. With the recent interest in GenAI, we can recognize that knowledge resides outside of humans and in this increasingly connected world, solutions to problems are provided by teams from multiple disciplines. GenAI can be used to provide personalized learning through communities, fostering a greater sense of autonomy and motivation [28,37].

If academia fails to embrace a connectivist framework, it risks losing its position as an arbiter of information while becoming less relevant.

The application of connectivism helps expand our approach to constructing scientific knowledge. It can move us from traditional classrooms to virtual communities by implementing solutions that are responsive, agile, networked, and dynamic [26]. When faced with emergent challenges with the rapid growth of information from multiple disciplines, Connectivism can bolster a swift response. We envision several applications of connectivism through social learning platforms that already exist for students to solve complex problems using online open-access tutoring through self-motivation [38]. Community-driven GenAI solutions already exist connecting 5 million users to computing resources for learning with open access to the latest technologies [39,40] with the potential to offer personalized learning and cloud-based integrations of GenAI for smart classrooms [41,42]. These social learning approaches demonstrate that connectivism directs the learner to a socially shared source of knowledge. The idea of distributed cognitive theory proposed by Hutchins [43] is relevant to connectivism in that it provides the framework and approach to lifelong and self-determined knowledge acquisition in social learning contexts

A limitation of connectivism is the overwhelming amount of information and diversity of opinions. Educators should address this by organizing the content by highlighting the common themes through coherence [29]. Another solution is teaching learners to identify the credibility and cognitive authority of web-based resources [44,45]. Teachers can tailor the lesson to the individual needs of learners, and consistent use of internet-based tools across lessons improves the learner’s confidence and learning ability to use these tools [46]. We provide a practical guide for the application of connectivism in Table 2. We highlight the need for understanding before applying its principles in real-world contexts by creating feeds and recognizing sources of information. Another solution to information overload is the use of effective filters for feeds and autogenerated information. Education around using social media and digital scholarship is still being defined and will need refinement and guidance from educators, although connectivism, at its core, should be self-directed by the learner.

**Table 2:** Learning activities for teaching connectivism and its application.

Course objectives	Learning objectives	Learning activities	How can it be incorporated into the curriculum?
Connectivism framework	Summarize principles of connectivism Compare the relevance of connectivism to other frameworks for learning about GenAIa Identify valid resources from misinformation	Create a session on GenAI; as prework, provide the seminal paper by Siemens (Connectivism: A Learning Theory for the Digital Age) [24] At the session, review the paper in a large group discussion Small group discussion on how connectivism is relevant for learning about and staying up to date on GenAI	Identify a learning community Identify experts on social media platforms Discuss a preprint article on GenAI [47] Describe the cognitive authority of communities or experts
GenAI concerns, limitations, and potential benefits.	List limitations, concerns, and benefits of GenAI Recognize settings when it is most applicable	Discussion board on the dangers of misinformation and how to avoid it Small group discussion on the dangers of not exploring diverse opinions	Provide an article on droplet transmission [9] Provide resources on scientific community sites that show
Practicing connectivism	Review a strategy for building, curating, and navigating a knowledge network Practice tools for implementing this strategy Discuss bias in GenAI	Provide tasks for identifying reliable sources of information. Identify tools for knowledge curation	Identify a list of reputable experts and reliable media articles [13] Identify expertise by looking at their track record for accuracy: explore institutional affiliations Track Google Scholar and PubMed citations of experts; explore other experts they follow Populate the list with people of multiple perspectives and disciplines Identify resources to address misinformation [48]

^a^GenAI: generative artificial intelligence.

## Discussion and Conclusion

In this commentary, we use a personal narrative to make the case for rethinking our approach to learning and acquiring knowledge in a connected world. We recognize the challenges posed by GenAI and the need for innovative solutions using connectivist approaches. Some of the challenges with GenAI are the concerns about biases, inaccuracies with hallucinations, and the accessibility of these tools. Being connected to learning communities, subscribing to credible and accurate sources of information, and being able to critically analyze information are potential solutions. Using freemium GenAI tools addresses some of the problems with accessibility and unfamiliarity with these tools. Embracing digital technologies and internet tools as health professional educators enables us to direct our learners through cohesive approaches to trainees to build, curate, and navigate knowledge networks that will help them address challenges with misinformation. As the amount of information and misinformation grows, one of the essential skills for health care practitioners will be the need to decipher accurate information from misinformation, which is possible by belonging to learning communities that amplify accurate information and expose the dangers of misinformation. GenAI poses an exciting opportunity to democratize resources through open-source collaborative efforts.

GenAI can be used across different learning paradigms, enabling learner-directed learning and empowering the teacher in a connectivist learning environment [49]. This approach of using GenAI as part of a new paradigm of learning allows educators to use these tools to provide powerful tutoring and adaptive testing based on analytics and to deliver personalized learning, improving assessment and feedback [50].

## Summary

### Practical Steps for Implementation in Health Care Education and Practice

First, creating a dynamic learning network encourages health care professionals and students to build and maintain professional networks through platforms like LinkedIn, X, and specialty-specific forums. Individuals can access real-time updates and exchange information. Use the feed generators from online communities and interested studies to build a knowledge network. Demonstrate the use of desired feed from journals on topics of interest. Identify experts from various disciplines.

Second, integrate technology into learning and practice; demonstrate the use of available resources such as podcasts, online webinars, blogs, and community forums to help with knowledge-building.

Third, develop critical information literacy skills, including training on how to critically assess the reliability and credibility of information sources, especially those found online. It is essential for learners to be able to distinguish between valid and misleading information and address misinformation.

Fourth, by leveraging artificial intelligence capabilities, clinicians can filter out unnecessary information, and GenAI tools can identify relevant scientific articles through feeds from reliable sources of information and amplify their search using freemium tools such as Research Rabbit. In addition, these tools allow for adaptive personalized instruction to the learner.

Fifth, the clinician can incorporate these tools as part of a synchronous classroom activity and model critical inquiry skills when using GenAI. By analyzing the accuracy of outputs, clinicians can model iterative prompting to refine GenAI outputs, refine learning, and expose the gaps in outputs.

## Abbreviations

GenAI: generative artificial intelligence

## References

[1] Gladiss Merlin NR, Jacobsen KH (2020). Statewide COVID-19 stay-at-home orders and population mobility in the United States. World Med Health Policy.

[2] Santarpia JL, Rivera DN, Herrera VL, Morwitzer MJ, Creager HM, Santarpia GW, Crown KK, Brett-Major DM, Schnaubelt ER, Broadhurst MJ, Lawler JV, Reid SP, Lowe JJ Transmission potential of SARS-CoV-2 in viral shedding observed at the University of Nebraska Medical Center. medRxiv. Posted online June 3, 2020.

[3] @linseycmarr. Let's talk about #airborne transmission of #SARSCOV2 and other viruses. A discussion is needed to improve accuracy and reduce fear associated with the term.

[4] Morawska L, Milton DK (2020). It is time to address airborne transmission of coronavirus disease 2019 (COVID-19). Clin Infect Dis.

[5] Randall K, Ewing ET, Marr LC, Jimenez JL, Bourouiba L (2021). How did we get here: what are droplets and aerosols and how far do they go? A historical perspective on the transmission of respiratory infectious diseases. Interface Focus.

[6] CDC statement for healthcare personnel on hand hygiene during the response to the international emergence of COVID-19. US Centers for Disease Control and Prevention.

[7] (2020). @WHO. FACT: #COVID19 is NOT airborne. The #coronavirus is mainly transmitted through droplets generated when an infected person coughs, sneezes or speaks.

[8] Jimenez JL, Marr LC, Randall K, Ewing ET, Tufekci Z, Greenhalgh T, Tellier R, Tang JW, Li Y, Morawska L, Mesiano-Crookston J, Fisman D, Hegarty O, Dancer SJ, Bluyssen PM, Buonanno G, Loomans MGLC, Bahnfleth WP, Yao M, Sekhar C, Wargocki P, Melikov AK, Prather KA (2022). What were the historical reasons for the resistance to recognizing airborne transmission during the COVID-19 pandemic?. Indoor Air.

[9] Greenhalgh T, Jimenez JL, Prather KA, Tufekci Z, Fisman D, Schooley R (2021). Ten scientific reasons in support of airborne transmission of SARS-CoV-2. Lancet.

[10] Modes of transmission of virus causing COVID-19: implications for IPC precaution recommendations. World Health Organization.

[11] Xie X, Li Y, Chwang ATY, Ho PL, Seto WH (2007). How far droplets can move in indoor environments--revisiting the Wells evaporation-falling curve. Indoor Air.

[12] Morawska L, Bahnfleth W, Bluyssen P, Boerstra A, Buonanno G, Dancer S, Floto A, Franchimon F, Haworth C, Hogeling J, Isaxon C, Jimenez JL, Kurnitski J, Li Y, Loomans M, Marks G, Marr LC, Mazzarella L, Melikov A, Miller S, Milton D, Nazaroff W, Nielsen PV, Noakes C, Peccia J, Querol X, Sekhar C, Seppänen Olli, Tanabe SI, Tellier R, Wai TK, Wargocki P, Wierzbicka A (2023). Coronavirus disease 2019 and airborne transmission: science rejected, lives lost. Can society do better?. Clin Infect Dis.

[13] The 60-year-old scientific screwup that helped covid kill. WIRED.

[14] Chapin CV (1912). The Sources and Modes of Infection.

[15] Woo PCY, Lau SKP, Chu C, Chan K, Tsoi H, Huang Y, Wong BHL, Poon RWS, Cai JJ, Luk W, Poon LLM, Wong SSY, Guan Y, Peiris JSM, Yuen KY (2005). Characterization and complete genome sequence of a novel coronavirus, coronavirus HKU1, from patients with pneumonia. J Virol.

[16] Yu IT S, Li Y, Wong TW, Tam W, Chan AT, Lee JHW, Leung DYC, Ho T (2004). Evidence of airborne transmission of the severe acute respiratory syndrome virus. N Engl J Med.

[17] Hare R (1964). The transmission of respiratory infections. Proc R Soc Med.

[18] Lewis D (2022). Why the WHO took two years to say COVID is airborne. Nature.

[19] Janis IL (1982). Groupthink: Psychological Studies of Policy Decisions and Fiascoes, 2nd Ed.

[20] Chapman SJ, Shelton B, Mahmood H, Fitzgerald JE, Harrison EM, Bhangu A (2014). Discontinuation and non-publication of surgical randomised controlled trials: observational study. BMJ.

[21] Kasenda B, von Elm E, You J, Blümle Anette, Tomonaga Y, Saccilotto R, Amstutz A, Bengough T, Meerpohl JJ, Stegert M, Tikkinen KAO, Neumann I, Carrasco-Labra A, Faulhaber M, Mulla SM, Mertz D, Akl EA, Bassler D, Busse JW, Ferreira-González Ignacio, Lamontagne F, Nordmann A, Gloy V, Raatz H, Moja L, Rosenthal R, Ebrahim S, Schandelmaier S, Xin S, Vandvik PO, Johnston BC, Walter MA, Burnand B, Schwenkglenks M, Hemkens LG, Bucher HC, Guyatt GH, Briel M (2014). Prevalence, characteristics, and publication of discontinued randomized trials. JAMA.

[22] Janis IL (1972). Victims of Groupthink: A Psychological Study of Foreign Policy Decisions and Fiascoes.

[23] Horgan D (2017). Building eminence through evidence. Biomed Hub.

[24] Siemens G (2004). Connectivism. Pressbooks.

[25] Brandao P, Algarvio D (2020). Connectivism, information technologies and distance learning. Kriativ.tech.

[26] Omodan BI, Mtsi N, Mpiti PT (2023). Enhancing virtual teaching and learning through connectivism in university classrooms. J Curric Teach.

[27] Goldie JGS (2016). Connectivism: a knowledge learning theory for the digital age?. Med Teach.

[28] Wongwatkit C, Thongsibsong N, Chomngern T, Thavorn S (122). The future of connectivist learning with the potential of emerging technologies and AI in Thailand: trends, applications, and challenges in shaping education. J Learn Sci Educ.

[29] Downes S (2020). Recent work in connectivism. Eur J Open Distance E-Learning.

[30] Omodan BI (2023). Analysis of connectivism as a tool for posthuman university classrooms. J Curric Stud Res.

[31] AlDahdouh A, Osorio A, Caires S (2015). Understanding knowledge network, learning and connectivism. Int J Instruct Tech Distance Learn.

[32] Lacković N (2020). Thinking with digital images in the post-truth era: a method in critical media literacy. Postdigit Sci Educ.

[33] Corbett F, Spinello E (2020). Connectivism and leadership: harnessing a learning theory for the digital age to redefine leadership in the twenty-first century. Heliyon.

[34] Utecht J, Keller D (2019). Becoming relevant again: applying connectivism learning theory to today's classrooms. Crit Quest Educ Acad Educ Stud.

[35] Hendricks GP (2019). Connectivism as a learning theory and its relation to open distance education. Progressio.

[36] Cleary Y (2020). Fostering communities of inquiry and connectivism in online technical communication programs and courses. J Tech Writ Commun.

[37] Fancsali SE, Holstein K, Sandbothe M, Ritter S, McLaren BM, Aleven V, Bittencourt II, Cukurova M, Muldner K, Luckin R, Millán E (2020). Towards practical detection of unproductive struggle. Artificial Intelligence in Education.

[38] Dautov R, Husom EJ, Sen S, Song H (2023). Towards community-driven generative AI. Ann Comp Sci Info Sys.

[39] Anderson DP, Cobb J, Korpela E, Lebofsky M, Werthimer D (2002). SETI@home. Commun ACM.

[40] Faritha Banu J, Revathi R, Suganya M, Gladiss Merlin NR (2020). IoT based cloud integrated smart classroom for smart and a sustainable campus. Procedia Comp Sci.

[41] Shemshack A, Spector JM (2020). A systematic literature review of personalized learning terms. Smart Learn Environ.

[42] Hutchins E Distributed Cognition. Cornell University.

[43] Fritch JW, Cromwell RL (2001). Evaluating Internet resources: Identity, affiliation, and cognitive authority in a networked world. J Am Soc Inf Sci.

[44] Kapoun J (2020). Teaching undergrads WEB evaluation: a guide for library instruction. C&RL News.

[45] Jeong H, Hmelo-Silver Ce (2010). Productive use of learning resources in an online problem-based learning environment. Comput Hum Behav.

[46] Wang T, Lund BD, Marengo A, Pagano A, Mannuru NR, Teel ZA, Pange J (2023). Exploring the potential impact of artificial intelligence (AI) on international students in higher education: generative AI, chatbots, analytics, and international student success. Appl Sci.

[47] Confronting medical misinformation: Tips from the trenches. Association of American Medical Colleges.

[48] Ouyang F, Jiao P (2021). Artificial intelligence in education: the three paradigms. Comput Educ Artif Intell.

[49] Choi E, Borkowski M, Zakoian J, Sagan K, Scholla K, Ponti C, Labedz M, Bielski M (2016). Proc Assoc Info Sci Tech.

[50] Al-Zahrani A, Alasmari TM (2024). Exploring the impact of artificial intelligence on higher education: the dynamics of ethical, social, and educational implications. Humanit Soc Sci Commun.

